# Music Form but Not Music Experience Modulates Motor Cortical Activity in Response to Novel Music

**DOI:** 10.3389/fnhum.2020.00127

**Published:** 2020-04-16

**Authors:** Patricia Izbicki, Andrew Zaman, Elizabeth L. Stegemöller

**Affiliations:** Department of Kinesiology, Iowa State University, Ames, IA, United States

**Keywords:** motor cortical excitability, music listening, music training, musicians and non-musicians, music experience

## Abstract

External cues, such as music, improve movement performance in persons with Parkinson’s disease. However, research examining the motor cortical mechanisms by which this occurs is lacking. Research using electroencephalography in healthy young adults has revealed that moving to music can modulate motor cortical activity. Moreover, motor cortical activity is further influenced by music experience. It remains unknown whether these effects extend to corticomotor excitability. Therefore, the primary aim of this study was to determine the effects of novel music on corticomotor excitability using transcranial magnetic stimulation (TMS) in a pilot study of healthy young adults. A secondary aim of this study was to determine the influence of music experience on corticomotor excitability. We hypothesized that corticomotor excitability will change during music conditions, and that it will differ in those with formal music training. Motor evoked potentials (MEPs) were recorded from the first dorsal interosseous using single-pulse TMS in three conditions: (1) No Music, (2) Music Condition I, and (3) Music Condition II. Both pieces were set to novel MIDI piano instrumentation and part-writing conventions typical of early nineteenth-century Western classical practices. Results revealed Music Condition II (i.e., more relaxing music) compared to rest increased MEP amplitude (i.e., corticomotor excitability). Music Condition II as compared to Music Condition I (i.e., more activating music) reduced MEP variability (i.e., corticomotor variability). Finally, years of formal music training did not significantly influence corticomotor excitability while listening to music. Overall, results revealed that unfamiliar music modulates motor cortical excitability but is dependent upon the form of music and possibly music preference. These results will be used to inform planned studies in healthy older adults and people with Parkinson’s disease.

## Introduction

There is increased interest in the effects and efficacy of using music to improve movement in neurodegenerative disorders, specifically Parkinson’s disease (PD). Dance, a combination of music and movement, has shown to improve mobility, gait, and postural instability in persons with PD (Hackney and Earhart, 2010; Foster et al., 2013; Houston and McGill, 2013; Volpe et al., 2013). Music listening and music therapy have been shown to improve motor performance in persons with PD (Sihvonen et al., 2017). However, it is still unclear how music impacts motor cortical activity. An understanding of the basic mechanisms of how music affects motor cortical activity in healthy young adults provides the foundation for further examination of how music influences movement in healthy older adults and persons with PD.

The phenomenon of music eliciting movement is present in humans and other species, suggesting an evolutionarily conserved trait (Patel et al., 2009). Studies have indicated that listening to music globally activates the cerebral cortex (Menon and Levitin, 2005; Bengtsson et al., 2009). More specifically, motor regions, including the primary motor cortex, supplementary motor area, pre-motor cortex, and basal ganglia, are involved in listening to music (Popescu et al., 2004; Baumgartner et al., 2007; Chen et al., 2008; Bengtsson et al., 2009). Thus, music seems to elicit movement through the coupling of sensorimotor processes in the brain (Janata et al., 2012), suggesting that music may be a tool to modulate motor cortical excitability.

Behavioral studies have shown faster tempo, moderate syncopation, and repetitive rhythm elicit a greater urge to move (i.e., high groove) while slower tempo, excessive syncopation, and non-repetitive rhythm elicit little to no urge to move (Janata et al., 2012; Witek et al., 2014). This suggests that different forms of music may have differential effects on motor cortical activity. Furukawa et al. (2017) have shown that even listening to different piano tones increases somatotopic specific motor cortical excitability when compared to listening to noise in musicians. While a tone does not encompass the complexity of a musical excerpt or represent a change in musical form, this study (along with previous studies) supports the notion that musical form may modulate motor cortical excitability.

Music expertise has also been shown to influence motor cortical activity (Koeneke et al., 2006). Changes in motor cortical plasticity have occurred in both short- and long-term piano learning (Bangert and Altenmüller, 2003). Furthermore, music experience has been shown to play a role in modulating motor cortical activity in response to music. Individuals with previous formal music training have shown greater motor cortical activity as compared to non-musicians while listening to previously learned music (Haueisen and Knösche, 2001). Listening to different piano tones demonstrated increased somatotopic specific motor cortical excitability in musicians but not non-musicians (Furukawa et al., 2017). A recent study using transcranial magnetic stimulation (TMS) while listening to familiar music has also shown that music modulates corticomotor excitability in both musicians and non-musicians (Stupacher et al., 2013). Thus, music listening modulates corticospinal excitability differently between musicians and non-musicians. However, these previous studies used familiar music or learned music. D’Ausilio et al. (2006) have been the only group (to our knowledge) to show that there is increased motor cortical excitability for non-rehearsed or “previously unheard” music in amateur piano players. How changes in motor cortical excitability differ between musicians and non-musicians while listening to novel music remains limited.

A meta-analysis using the activation likelihood estimation approach found that music familiarity increased audio-motor synchronization to rhythm in familiar music vs. unfamiliar music (Freitas et al., 2018). In addition, a recent study has been conducted examining motor cortical activity in response to previously novel music using electroencephalography (EEG). Results found differential responses to musical form over the sensorimotor cortex that was further influenced by music experience, which may be reflective of a decrease in movement variability (Stegemöller et al., 2018a). However, EEG cannot determine specific neuronal activity (i.e., excitability). TMS is a technique that can determine more specific neuronal activity and variability in the motor cortex via motor evoked potential (MEP) amplitude and MEP variability. Previous research has shown that MEP amplitude is inversely related to MEP variability (Kiers et al., 1993; Devanne et al., 1997; Darling et al., 2006) Furthermore, exposure to sensory stimuli (e.g., visual, auditory, olfactory) have been shown to modulate MEP amplitude and/or variability (Furubayashi et al., 2000; Carson et al., 2005; Rossi et al., 2008).

Thus, the aim of this study was to determine the effects of listening to two novel musical pieces on motor cortical excitability of the hand area in the primary motor cortex using TMS. We hypothesized that both pieces will increase motor cortical excitability of the hand area, as measured by motor evoked potential. A second aim of this study was to determine the influence of previous music experience on motor cortical excitability. We hypothesized that motor cortical excitability of the hand area will be different for musicians than non-musicians.

## Materials and Methods

### Participants

Twenty healthy young adults were recruited (11 women, mean age ± standard deviation age = 21 ± 2.03). See Table 1 for detailed demographic information. All participants provided written informed consent to participate in the study as approved by the university Institutional Review Board. All procedures performed in studies involving human participants were in accordance with the ethical standards of the institution and with the 1964 Helsinki declaration and its later amendments or comparable ethical standards. Inclusion criteria included only healthy young adults between ages 18–40. Exclusion criteria included significant cognitive impairment (Mini Mental State Exam (MMSE) <24) and/or major depression (Beck Depression Inventory (BDI) >18). Exclusion criteria for TMS included any previous adverse reactions to TMS, previous seizure, surgery on blood vessels, brain, or heart, previous stroke, severe vision or hearing loss, metal in head, implanted devices, severe headaches, previous brain-related conditions, brain injury, medications (i.e., antibiotics, antifungal, antiviral, antidepressants, antipsychotics, chemotherapy, amphetamines, bronchodilators, anticholinergics, antihistamines, sympathomimetics), family history of epilepsy, pregnancy, alcohol consumption less than 24 h before study, smoking, and illicit drug use.

**TABLE 1:** Participant demographics and music experience.

**Demographics**	**Musician**	**Non-musician**
Age (Mean ± SD)	21 ± 1.4	22 ± 2.6
Gender (%Male, %Female)	50%, 50%	40%, 60%
Ethnicity (%Caucasian, %Hispanic, %Asian	80%, 20%, 0%	90%, 0%, 10%
Handedness (%RH, %LH)	90%, 10%	100%
Music training (Years)	9.8 ± 4.1	1.0 ± 1.8
Instrument (%)	10% Clarinet, 50% Piano, 10% Trumpet, 10% Viola, 20% Voice	10% Bass, 10% Saxophone, 0% Voice, 70% NA

*SD, standard deviation; RH, right hand; LH, left hand.*

### Participant Music Experience

Prior to TMS data collection, all participants orally provided information about their previous music experience. The researchers asked participants to provide total years of formal music training and instrument played. Formal music training was defined as private music lessons on an instrument or voice. Participants were classified as musicians (≥5 years experience, *n* = 10, mean ± standard deviation = 9.8 ± 4.1 years) or non-musicians (<5 years experience, *n* = 10, mean ± standard deviation = 1.0 ± 1.7 years). See Table 1 for detailed information on music experience.

Five years of formal music training was chosen as a cutoff because healthy young adult participants who had more than 5 years of music experience received advanced training (i.e., late middle school, high school, and collegiate level). Furthermore, other studies in children and adult musicians have characterized music experience groups using the number of years of music training (Wong et al., 2007; Hanna-Pladdy and MacKay, 2011; Stegemöller et al., 2018a; Strong and Mast, 2018).

### Music

The music was specifically commissioned for the study by an (Iowa State University) music composition student in order to control for previous experience or familiarization with the music. Both pieces were set to novel MIDI piano instrumentation and part-writing conventions typical of early nineteenth-century Western classical practices. Music Condition I was set in the key of C major in ternary form (ABA’), 4/4 meter, and a quarter note pulse of 140 beats per minute (BPM). Music Condition II was set in the key of G-flat major in through-composed form, 3/4 meter, and a quarter note pulse of 70 beats per minute. The piece contained greater tonal and rhythmic variations than Music Condition I. These are the same pieces that were used in a previous EEG study (see Supplementary Material) (Stegemöller et al., 2018b). Participants were asked their preference for the two forms of music based on a Likert scale of 1–10. 1 indicated the participants extremely disliked the form and 10 indicated the participants extremely liked the music.

### Data Collection

For TMS, the motor hot spot, specifically the hand knob area in the primary motor cortex (M1), was located on the contralateral hemisphere (left hemisphere; all participants were right-handed). The location and coil orientation (45 degrees to the left of the longitudinal fissure) was marked, and the coil was held in a constant position by the experimenter with the aid of a coil holder. Resting motor threshold (RMT) (i.e., a MEP at an amplitude of at least 50 μV produced for 5 out of 10 trials or 50% of the time) was then found. RMT was completed in 30 min. Single-pulse TMS intensity was set at 120% of RMT.

Participants were seated in an armchair with their right forearm pronated and rested on the armrest. Participants were asked not to move during TMS. Single-pulse TMS was applied to the M1 dominant hand area using the Magstim Model 200 (Magstim, Whitland, Carmarthenshire). The coil was figure-8 coil (7 cm outer diameter of wings). Coil current was induced approximately perpendicular to the motor homunculus and central sulcus. The waveform was monophasic. Spike2 was used to trigger single-pulse stimulations via a Power 1401 data acquisition board and Spike2 software (Cambridge Electronic Design (CED), Cambridge, United Kingdom). Motor evoked potentials (MEPs) were recorded from the right first dorsal interosseous (FDI) using bipolar surface electromyography (EMG) (Delsys, Boston, MA, United States). Twenty single-pulse stimulations were applied during rest (no music) and while passively and continuously listening to two different music selections. The total number of pulses applied across all conditions was 60. There was 5 min of rest (no TMS) between each stimulation condition. Single-pulses were applied approximately every 5 s (for a total of 1.7 min of stimulation in each condition) and were not specifically timed to the beat of the music. Each non-music and music condition lasted 5 min. Along with the informed consent process, the entire experiment lasted around an hour. The order of the music selections was randomized between participants, and TMS was applied during random sections of each music selection.

### Data Analysis

EMG signals were notch filtered (60 Hz) and high-pass filtered (2nd-order dual-pass Butterworth, 2 Hz cut-off). EMG signals were also DC shifted, and the root mean square of the EMG signal was obtained. Peak-to-peak amplitude (μV) was obtained within 100 ms of the TMS pulse. Background EMG was determined for periods of 1.25–0.25s before the peak maximum amplitude and 0.25–1.25s after the peak maximum amplitude. Background EMG trials > 10 μV were discarded (Majid et al., 2015). For EMG activity before peak amplitude, the number of trials discarded were 8 trials in the rest condition, 0 trials in the Music Condition I, and 15 trials in the Music Condition II. For EMG activity after peak amplitude, the number of trials discarded were 5 trials in the rest condition, 1 trial in the Music Condition I, and 15 trials in the Music Condition II. The raw data for each participant in the background EMG activity and for each condition was natural log transformed to obtain a normal distribution. The primary outcome measure of MEP amplitude was obtained by averaging the natural log transformed MEP trials for each condition (i.e., No Music, Music Condition I, and Music Condition II) in the stimulation parameter (i.e., single-pulse) (Nielsen, 1996; Clark et al., 2004). Coefficient of variation (CV) (standard deviation divided by average) was calculated for each participant in each condition. CV was used as the MEP variability measure (Klein-Flügge et al., 2013).

### Statistical Analysis

Statistical analysis was completed in IBM SPSS Statistics for Windows, Version 25.0 (IBM Corp., Armonk, NY, United States). Normality was assessed using the Shapiro-Wilk test. Analyses were completed to determine if there was any potential influence of music preference and background EMG activity on the main outcome measures. Due to the non-normality of the music preference data, a Wilcoxon signed rank test was used to compare whether there was any overall difference in preference to Music Condition I vs. Music Condition II overall. The Mann-Whitney U test was used to compare whether there were any differences in music preference in musicians vs. non-musicians. Due to the non-normality of the background EMG activity pre- and post- MEP, the Friedman test was conducted to determine differences in EMG background activity among all conditions for both pre-MEP EMG activity and post-MEP EMG activity. To examine differences in peak maximum amplitude of the MEP between the three music conditions, a (two-way) mixed ANOVA was completed. The within factor was music condition (Rest, Music Condition I, and Music Condition II) while the between factor was musician or non-musician. To examine the influence of musical form and music training on MEP amplitude and variability (CV), a (two-way) mixed ANOVA was completed. The within factor was music condition (Rest, Music Condition I, Music Condition II) while the between factor was musician or non-musician. Bonferroni correction was used for *post- hoc* analysis. Significance was set at α = 0.05.

## Results

### Music Preference

**FIGURE 1:**
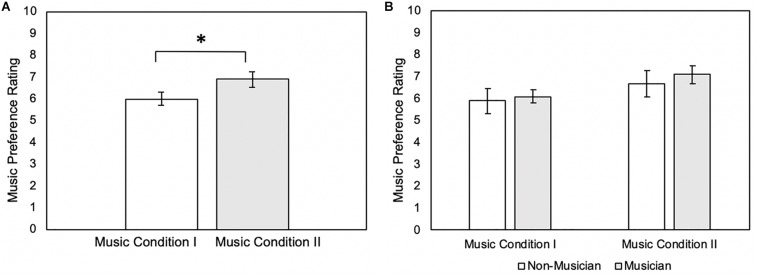
**(A)** Music preference rating of Music Condition I and Music Condition II (*N* = 20). Error bars reflect standard error of mean music preference ratings. *p = 0.007. **(B)** Music preference rating of Music Condition I and Music Condition II between non-musicians (*N* = 10) and musicians (*N* = 10). Error bars reflect standard error of mean music preference ratings.

The Wilcoxon signed-rank test showed that participant ratings for Music Condition II were significantly larger than for Music Condition I (*Z* = −2.68, *p* = 0.007) (mean ± standard deviation: Music Condition I = 5.95 ± 1.32; Music Condition II = 6.90 ± 1.48) (Figure 1A and Table 2). However, the Mann-Whitney U test showed no significant differences in music preference between musicians and non-musicians for Music Condition I (*U* = 43.5, *p* = 0.612) (mean ± standard deviation: musicians Music Condition I = 6.10 ± 0.994; non-musicians Music Condition I = 5.8 ± 1.62) and Music Condition II (*U* = 43.0, *p* = 0.577) (mean ± standard deviation: musicians Music Condition II = 7.10 ± 1.29; non-musicians Music Condition II = 6.70 ± 1.70) (Figure 1B and Table 2).

**TABLE 2:** Means and standard deviations for music preference.

**Condition**	**Total**	**Musicians**	**Non-musicians**
Music Condition I	5.95 ± 1.32	6.10 ± 0.994	5.8 ± 1.62
Music Condition II	6.90 ± 1.48	7.10 ± 1.29	6.70 ± 1.70

### Background EMG

**FIGURE 2:**
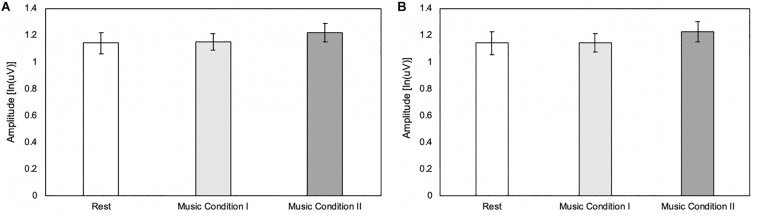
**(A)** Pre-stimulation background EMG 1.25–0.25s before the peak maximum amplitude of the MEP (*N* = 20). Error bars reflect standard error of mean EMG activity. **(B)** Post-stimulation background EMG 0.25–1.25s after the peak maximum amplitude of the MEP (*N* = 20). Error bars reflect standard error of mean EMG activity.

To confirm that potential differences in MEP amplitude are due to cortical mechanisms rather than an increase in drive to spinal mechanisms, a Friedman test was conducted to compare 1.25 to 0.25s before the peak maximum amplitude among the three conditions as well as 0.25 to 1.25s after the peak maximum amplitude among the three conditions. Results revealed no differences in EMG activity before [χ^2(2)^ = 4.80, *p* = 0.091] or after [χ^2(2)^ = 3.90, *p* = 0.142] peak maximum amplitude (Figures 2A,B).

### MEP Amplitude

**FIGURE 3:**
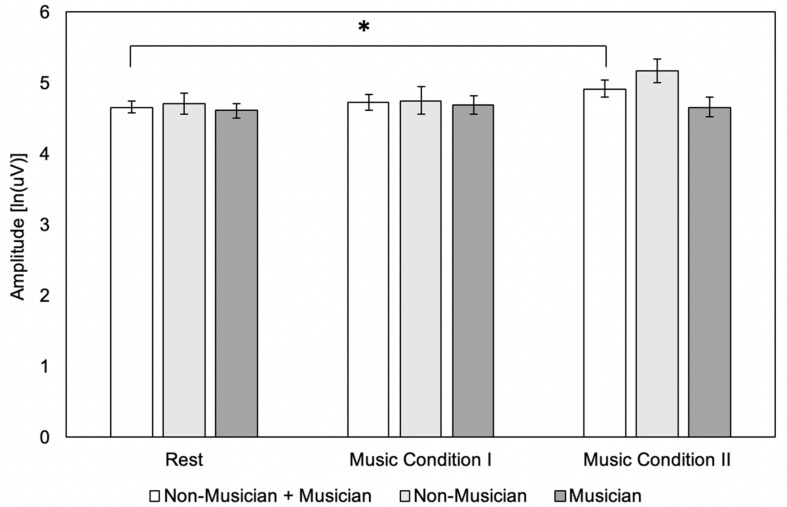
Motor evoked potential (MEP) amplitude between conditions (*N* = 20) and groups (*N* = 10) using the single-pulse paradigm (*N* = 20). Error bars reflect standard error. *p = 0.05.

There was a significant main effect of condition (*F*_(__2,36__)_ = 3.51, *p* = 0.04), but no significant main effect of group (*F*_(__1,18__)_ = 1.65, *p* = 0.22). There was no significant interaction effect (*F*_(__2,36__)_ = 3.15, *p* = 0.05). *Post hoc* tests using Bonferroni correction for the main effect of condition (*p* < 0.017) revealed that MEP amplitude did not differ for Music Condition I compared to rest (4.73 ± 0.51 vs. 4.66 ± 0.39 uV) (*p* = 1.00) or for Music Condition I compared to Music Condition II (4.73 ± 0.51 vs. 4.92 ± 0.54 uV) (*p* = 0.06). Music Condition II compared to rest revealed a significant increase in MEP amplitude (4.92 ± 0.54 vs. 4.66 ± 0.39 uV) (*p* = 0.017). *Post hoc* tests using Bonferroni correction for the interaction effect (*p* < 0.005) are listed in Table 3. Results revealed no significant differences in musicians for Music Condition I compared to rest, Music Condition I compared to Music Condition II, and Music Condition II compared to rest. Results revealed no significant differences in non-musicians for Music Condition I compared to rest, Music Condition I compared to Music Condition II, and Music Condition II compared to rest. Results revealed no significant differences in musicians and non-musicians for Rest, Music Condition I, or Music Condition II (Figure 3).

**TABLE 3:** *Post hoc* tests using bonferroni correction for the interaction effect (*p* < 0.005).

**Comparison**	**Mean ± Standard Deviation**	***P*-value**
Music Condition I vs. Rest (Musicians Only)	4.70 ± 0.41 vs. 4.62 ± 0.31 uV	0.38
Music Condition I vs. Music Condition II (Musicians Only)	4.70 ± 0.41 vs. 4.66 ± 0.45 uV	0.46
Music Condition II vs. Rest (Musicians Only)	4.66 ± 0.45 vs. 4.62 ± 0.31 uV	0.57
Music Condition I vs. Rest (Non-Musicians Only)	4.76 ± 0.61 vs. 4.71 ± 0.46 uV	0.83
Music Condition I vs. Music Condition II (Non-Musicians Only)	4.76 ± 0.61 vs. 5.18 ± 0.52 uV	0.05
Music Condition II vs. Rest (Non-Musicians Only)	5.18 ± 0.52 vs. 4.71 ± 0.46 uV	0.03
Musicians vs. Non-Musicians (Rest)	4.62 ± 0.31 vs. 4.71 ± 0.46 uV	0.50
Musicians vs. Non-Musicians (Music Condition I)	4.70 ± 0.41 vs. 4.76 ± 0.61 uV	0.79
Musicians vs. Non-Musicians (Music Condition II)	4.66 ± 0.45 vs. 5.18 ± 0.52 uV	0.03

### MEP Variability

**FIGURE 4:**
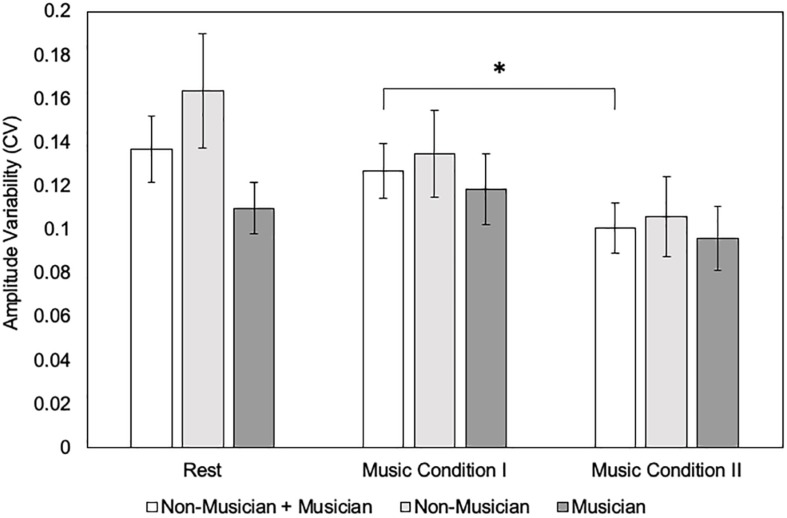
Motor evoked potential (MEP) amplitude variability (coefficient of variation = CV) between conditions (*N* = 20) and groups (*N* = 10) using the single-pulse paradigm. Error bars reflect standard error. *p = 0.05.

For MEP amplitude CV, results revealed a significant main effect of condition (*F*_(__2,36__)_ = 4.38, *p* = 0.02), but no significant main effect of group (*F*_(__1,18__)_ = 1.53, *p* = 0.23). There was no significant interaction effect (*F*_(__2,36__)_ = 1.79, *p* = 0.18). *Post hoc* tests using Bonferroni correction for the main effect (*p* < 0.017) revealed that MEP amplitude CV did not differ for the Music Condition I compared to rest (0.13 ± 0.057 vs. 0.13 ± 0.068 uV) (*p* = 1.00). Music Condition I compared to Music Condition II revealed a significant increase in MEP amplitude CV (0.13 ± 0.057 vs. 0.10 ± 0.051 uV) (*p* = 0.05). Music Condition II compared to rest did not reveal a significant difference in MEP amplitude CV (0.10 ± 0.051 vs. 0.13 ± 0.068 uV) (*p* = 0.06) (Figure 4).

## Discussion

The primary purpose of this study was to determine the effects of listening to two different forms of novel music on motor cortical excitability in the primary motor cortex using TMS. The secondary purpose of this study was to determine the influence of previous music experience on motor cortical excitability. We hypothesized that (1) motor cortical excitability of the hand area, as measured by motor evoked potential (MEP) amplitude, will differ between musical forms and (2) that both forms of music would increase MEP amplitude. Our findings partially support this hypothesis, revealing a main effect of condition. However, only Music Condition II differed from the rest condition. There was no difference between the two music conditions. For variability, a main effect of condition was also revealed, with *post hoc* analyses demonstrating a difference between the two music conditions. For our hypothesis regarding music experience, our findings did not support our hypothesis. No differences were revealed between musicians and non-musicians. To our knowledge, results from this study are the first to show that novel, preferred music selections may have a different motor cortical influence as compared to previous studies using familiar or learned music.

### MEP Amplitude

An interesting finding of this study was an increase in MEP amplitude for Music Condition II as compared to rest. Although participants in this study were not specifically asked if they perceived the music used in the study as relaxing or activating, music in Music Condition I was composed to evoke more of an activated feeling while music in Music Condition II was initially composed to evoke more of a relaxed feeling. Thus, the finding that Music Condition II resulted in an increase in motor cortical excitability seems contradictory to previous literature. Faster tempo, moderate syncopation, and repetitive rhythm have been shown to elicit a greater urge to move (i.e., high groove) while slower tempo, excessive syncopation, and non-repetitive rhythm elicit little to no urge to move (Janata et al., 2012). However, D’Ausilio et al. (2006) showed that there is increased motor cortical excitability for non-rehearsed or previously unheard music in amateur piano players. Additionally, Weigmann demonstrated that less predictable music (i.e., slightly more complex) generated more prediction errors which was reflected as greater pleasure and a greater urge to move. However, the rhythm must still be simple enough and not too complex to see this effect (Weigmann, 2017). This may be reflected in Music Condition II. Thus, the change in motor cortical excitability may be due to unfamiliarity as well as the wider range of rhythmic and harmonic variations found in Music Condition II regardless of the intended perception. This would suggest that future studies examining the effect of musical form on motor cortical activity should consider both participant perception and music composition.

Another consideration that may have influenced a difference in MEP amplitude for Music Condition II as compared to rest is the higher preference for the Music Condition II music than the Music Condition I in our sample. Listening to pleasurable music stimulates areas of the brain responsible for dopamine production (i.e., nucleus accumbens and ventral tegmental area) in both humans (Menon and Levitin, 2005) and rats (Moraes et al., 2018). These changes in dopamine have been implicated in modulating motor cortical activity (Ziemann et al., 1997; Jenkinson and Brown, 2011) as well as motor cortical plasticity (Calabresi et al., 2007; Molina-Luna et al., 2009). Thus, an increase in preference for Music Condition II may have increased dopamine production, which may modulate motor cortical activity resulting in increased motor cortical excitability of the hand area. However, no measures of dopamine were taken in this study leaving room for continued research to determine the relationship between preferred music, dopamine, and motor cortical activity.

### MEP Variability

An additional finding of this study revealed a decrease in the variability of motor cortical excitability while listening to Music Condition II compared to Music Condition I. This could be due to neural synchrony in motor cortical excitability. An increase in neural synchronization has been shown in individuals listening to music (Bernardi et al., 2017). This decrease in variability may transfer to movement performance. In a previous study from our lab, results revealed that repetitive finger movement variability significantly decreased while moving in time with music (Stegemöller et al., 2018a, b). The same two music samples as used in this study were used in this previous study. While the same participants were not tested, perhaps the decrease in MEP variability transfers to a decrease in movement variability. Future studies using TMS while moving with music are needed to confirm this notion. Nonetheless, this study provides continued evidence suggesting that music decreases variability of the motor system.

### MEP Amplitude and Variability in Musicians vs. Non-musicians

The final result of this study revealed no differences between musicians and non-musicians across all conditions. These results are in contrast to previous studies. Other studies have indicated increases in motor cortical excitability in musicians vs. non-musicians without listening to music (Rosenkranz et al., 2007) and while listening to high-groove music (Stupacher et al., 2013). However, there were no novel musical stimuli composed for each of the studies. A recent meta-analysis found familiar music elicited a greater motor pattern of activation as compared to unfamiliar music. Specifically, the ventral lateral nucleus (a motor first-order relay nucleus responsible for receiving input from substantia nigra, internal globus pallidus, and cerebellum) had the second highest likelihood for activation while listening to familiar music (Freitas et al., 2018). Furthermore, greater motor cortical activation for familiar music compared to unfamiliar music has been found in musicians (D’Ausilio et al., 2006). Thus, unfamiliarity with the musical stimuli in our study may have influenced the lack of difference in motor cortical excitability between musicians and non-musicians.

In short, our study is in keeping with previous literature on the neural basis of music familiarity. It may be that motor cortical differences are not dependent on musician/non-musician status but due to previous experience with a musical piece. This suggests that engagement with previously heard music may be beneficial for altering motor cortical activity. This has implications toward PD and music therapy, where people receiving music therapy are likely not musicians.

### Parkinson’s Disease and Motor Cortical Activity

The findings from our study have important implications for using music therapy and music and medicine interventions in persons with PD. Differences in beta band oscillations in the motor cortex have been shown in previous literature in persons with PD (Brown, 2007; Stegemöller et al., 2016, 2017). This indicates that motor cortical activity in persons with PD is different than in healthy older adults. In studies of motor cortical activity using TMS, drug-naïve patients have been shown to have increased MEPs at rest (Derejko et al., 2013). Although, music listening and music therapy have been shown to improve motor performance in persons with PD (Sihvonen et al., 2017), results of this study suggest that increasing motor cortical activity using certain music conditions may not necessarily be beneficial. On the other hand, decreasing the variability in the motor system with music, as demonstrated in this study, may be beneficial for persons with PD. Thus, as research on the underlying mechanisms of music therapy continues to grow, a clear understanding of music impacts the motor system in neurological populations is needed. This study provides continued information in understanding the impact of music on motor cortical excitability.

### Limitations

A limitation of this study was that there was no survey for perception of music (i.e., whether the music was relaxing, activating, and/or emotionally stimulating). However, the music used for each condition was distinctly different and represents two contrasting forms of music regardless of the form (relaxing or activating) perceived. In addition, TMS was applied at rest and not during movement. Given the tempi of the music, movements would have been completed at either 70 or 140 beats per minute. Completing repetitive finger movements at these rates while applying TMS can be done, but also increases the potential error in obtaining MEPs due to underlying muscle activity. Thus, applying TMS at rest in the various conditions was the initial first step in understanding how music influences motor cortical activity.

## Conclusion

In conclusion, results revealed that unfamiliar music modulates motor cortical excitability, but is dependent upon the form of music and possibly music preference. In addition, the form of music has a differing effect on motor cortical variability. However, there are no differences in motor cortical excitability between musicians and non-musicians when listening to unfamiliar music. These results suggest that music could be used to influence excitatory activity in the primary motor cortex and potentially reduce variability of the motor system regardless if a person is a musician or non-musician. This has implications toward PD and music therapy, where people receiving music therapy are likely not musicians. An understanding of the basic mechanisms of how music affects motor cortical activity in healthy young adults is needed to provide the foundation for further examination of how music influences movement in healthy older adults and persons with PD. Future studies will involve a similar paradigm with healthy older adults and people with Parkinson’s disease to further elucidate the influence of music on motor cortical activity in these populations.

## Data Availability Statement

The datasets generated for this study are available on request to the corresponding author.

## Ethics Statement

The studies involving human participants were reviewed and approved by the Iowa State University Institutional Review Board. The patients/participants provided their written informed consent to participate in this study.

## Author Contributions

PI and ES made substantial contributions to the concept and design of the study, acquisition of the data, analysis and interpretation of the data, and drafting and revising the article. AZ made contributions to analysis and interpretation of the data. All authors gave final approval of the version to be submitted.

## Conflict of Interest

The authors declare that the research was conducted in the absence of any commercial or financial relationships that could be construed as a potential conflict of interest.
