# Supplementary material for: Music Form but Not Music Experience Modulates Motor Cortical Activity in Response to Novel Music
Source: Front Hum Neurosci. 2020 Apr 16;14:127. doi: 10.3389/fnhum.2020.00127 (PMC7179827; doi:10.3389/fnhum.2020.00127)
Supplement: Supplementary file 1 [file Data_Sheet_1.PDF]

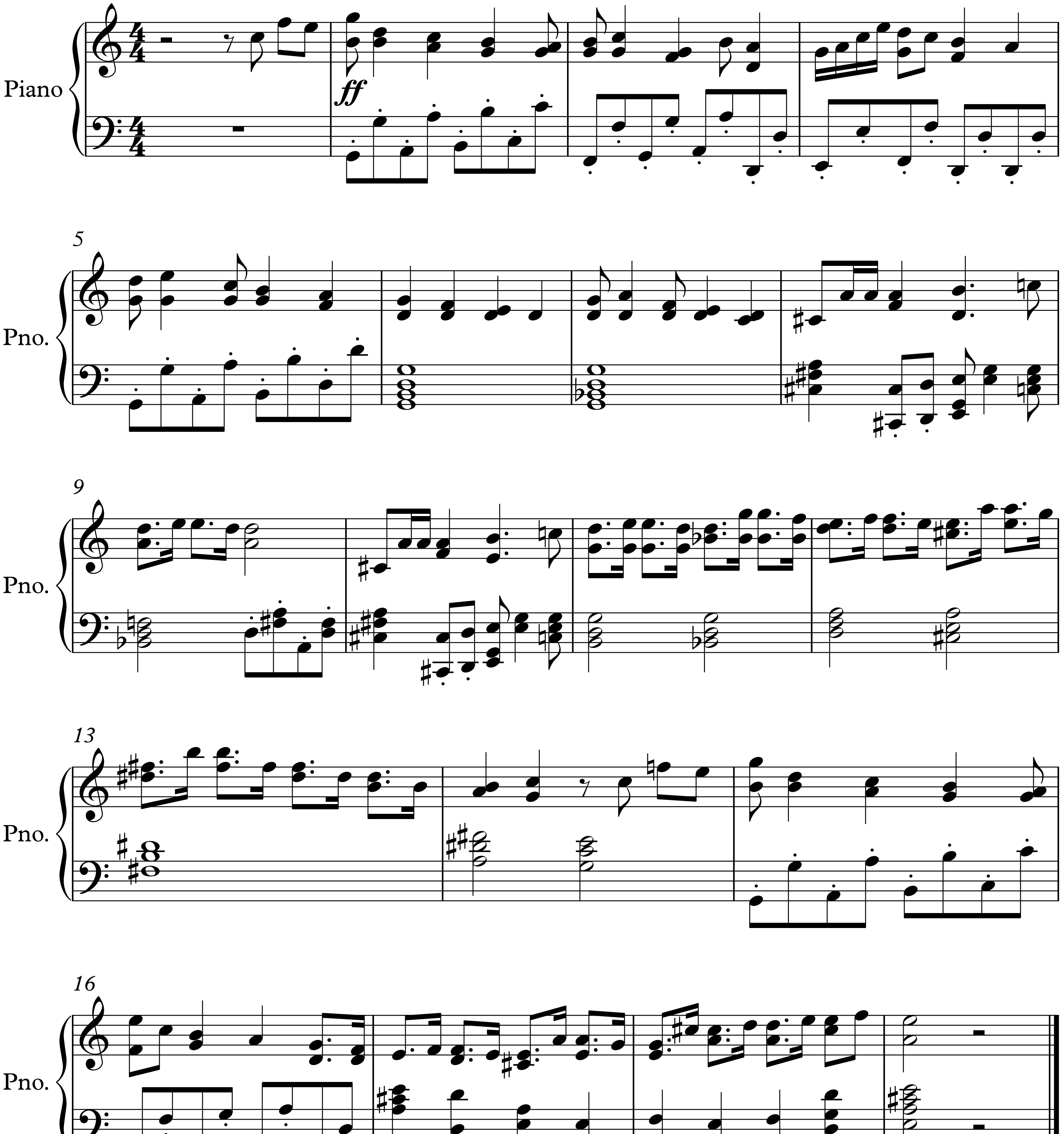
Piano
Pno.
Pno.
Pno.
Pno.
